# Effects of Sodium and Amino Acid Substrate Availability upon the Expression and Stability of the SNAT2 (SLC38A2) Amino Acid Transporter

**DOI:** 10.3389/fphar.2018.00063

**Published:** 2018-02-07

**Authors:** Thorsten M. Hoffmann, Emma Cwiklinski, Dinesh S. Shah, Clare Stretton, Russell Hyde, Peter M. Taylor, Harinder S. Hundal

**Affiliations:** Division of Cell Signalling and Immunology, Sir James Black Centre, School of Life Sciences, University of Dundee, Dundee, United Kingdom

**Keywords:** SNAT5, adaptive regulation, sodium ion, amino acid sensing, transporter, System A

## Abstract

The SNAT2 (SLC38A2) System A amino acid transporter mediates Na^+^-coupled cellular uptake of small neutral α-amino acids (AAs) and is extensively regulated in response to humoral and nutritional cues. Understanding the basis of such regulation is important given that AA uptake *via* SNAT2 has been linked to activation of mTORC1; a major controller of many important cellular processes including, for example, mRNA translation, lipid synthesis, and autophagy and whose dysregulation has been implicated in the development of cancer and conditions such as obesity and type 2 diabetes. Extracellular AA withdrawal induces an adaptive upregulation of SNAT2 gene transcription and SNAT2 protein stability but, as yet, the sensing mechanism(s) that initiate this response remain poorly understood although interactions between SNAT2 and its substrates may play a vital role. Herein, we have explored how changes in substrate (AA and Na^+^) availability impact upon the adaptive regulation of SNAT2 in HeLa cells. We show that while AA deprivation induces SNAT2 gene expression, this induction was not apparent if extracellular Na^+^ was removed during the AA withdrawal period. Furthermore, we show that the increase in SNAT2 protein stability associated with AA withdrawal is selectively repressed by provision of SNAT2 AA substrates (*N*-methylaminoisobutyric acid and glutamine), but not non-substrates. This stabilization and substrate-induced repression were critically dependent upon the cytoplasmic N-terminal tail of SNAT2 (containing lysyl residues which are putative targets of the ubiquitin-proteasome system), because “grafting” this tail onto SNAT5, a related SLC38 family member that does not exhibit adaptive regulation, confers substrate-induced changes in stability of the SNAT2-5 chimeric transporter. In contrast, expression of SNAT2 in which the N-terminal lysyl residues were mutated to alanine rendered the transporter stable and insensitive to substrate-induced changes in protein stability. Intriguingly, SNAT2 protein stability was dramatically reduced in the absence of extracellular Na^+^ irrespective of whether substrate AAs were present or absent. Our findings indicate that the presence of extracellular Na^+^ (and potentially its binding to SNAT2) may be crucial for not only sensing SNAT2 AA occupancy and consequently for initiating the adaptive response under AA insufficient conditions, but for enabling substrate-induced changes in SNAT2 protein stability.

## Introduction

The System A amino acid (AA) transporter family is comprised of three isoforms, SNAT1, 2, and 4, of which SNAT2 (SLC38A2) is the most widely expressed and most extensively regulated (Mackenzie and Erickson, 2004). SLC38 family members mediate the sodium ion-coupled uptake of short chain neutral AAs some of which (e.g., methionine and threonine) are considered nutritionally indispensable, whereas others (e.g., alanine and glutamine) are important for intermediary tissue metabolism and inter-organ flow of carbon and nitrogen (Hundal, 1991). Members of this carrier family exhibit tolerance for AAs with *N*-methyl substitutions such as *N*-methylaminoisobutyric acid (Me-AIB); a property that has proved useful in discriminating System A isoforms from other AA transporters and functionally characterizing the SLC38 family. SNAT2-mediated AA transport is highly pH sensitive (Baird et al., 2006), modulated by glucocorticoids and, in skeletal muscle cells, acutely stimulated by growth factors and insulin by a process that involves recruitment of intracellular SNAT2 molecules to the plasma membrane (Hundal et al., 1994; Hyde et al., 2002). Cellular expression/activity of SNAT2 is also elevated in response to hyperosmotic stress, which stimulates uptake of organic osmolytes (AAs) and establishes an osmotic gradient for water movement that helps normalize cell volume and reduce intracellular ionic strength (Bussolati et al., 2001; Lopez-Fontanals et al., 2003; Bevilacqua et al., 2005). The secondary active transport of extracellular AAs results in an outwardly directed concentration gradient of SNAT substrates that we have shown can subsequently exit the cell *via* tertiary exchange transporters, such as System L, which operate alongside SNAT carriers in the plasma membrane (Baird et al., 2009; Hundal and Taylor, 2009). This tertiary exchange coupling is of particular significance as it influences the intracellular delivery of essential AAs (e.g., leucine) that have a potent stimulatory effect on mTORC1, a signaling complex regulating key cellular processes such as mRNA translation, cell growth/metabolism, and autophagy (Saxton and Sabatini, 2017). Consequently, it follows that factors affecting expression and activity of SNAT2 may also impact on mTORC1 activation and regulation of these latter processes (Pinilla et al., 2011; Uno et al., 2015).

Another major feature of SNAT2 is its cellular upregulation in response to extracellular AA limitation (Kilberg et al., 1985; Guma et al., 1992; Hyde et al., 2001). Such upregulation is a property shared by a group of genes involved in AA biosynthesis (e.g., asparagine synthase) and transport and normally referred to as adaptive regulation. As such, this phenomenon involves the GCN2/ATF4 pathway, which helps coordinate a suppression in global protein synthesis and an increase in the expression of select transport proteins (e.g., SNAT2) that help facilitate recovery of cell volume and AAs once the availability of extracellular AAs has been restored (Kilberg et al., 2005). The transcriptional increase in SNAT2 gene expression is dependent upon an AA responsive domain in the first intron of the *SLC38A2* gene by ATF4 (Palii et al., 2006). However, in addition to the genomic-driven increase in SNAT2, we have previously shown that the adaptive increase in SNAT2 also involves enhanced stabilization of the SNAT2 protein (Hyde et al., 2007). This increase in stability is not seen for SNAT5, a structurally related transporter from the SLC38 gene family, which does not tolerate Me-AIB as a substrate or exhibit adaptive regulation. Strikingly, the increase in SNAT2 protein stability induced in response to AA withdrawal is associated with an isoform-specific regulatory domain(s) present within the hydrophilic N-terminal region, given that expression of a SNAT2–SNAT5 chimera in which the SNAT5 N-terminal domain is substituted with that of SNAT2 promotes stabilization of the chimeric (SNAT2-5) protein upon AA withdrawal (Hyde et al., 2007). Intriguingly, the functional increase in System A/SNAT transport activity seen in response to extracellular AA withdrawal can be repressed by resupply of any one single SNAT2 substrate AA even when all other AAs remain absent (Hyde et al., 2007). Since non-substrates do not exert this repressive effect on System A adaptation, the observations indicate that SNAT2 possesses a dual “transceptor” function in which occupancy of the SNAT2 substrate-binding site not only initiates transmembrane AA transfer but also enables sensing of AA sufficiency that is linked to regulation of SNAT2 expression and stability. We have previously demonstrated that single provision of SNAT2 substrate AAs to AA-starved cells represses the adaptive increase in SNAT2 gene promoter activity (Hyde et al., 2007), but hitherto it remains unknown whether substrate-induced repression of SNAT2 also entails reduced SNAT2 protein stability. In this study, we have explored the effects of substrate (AA and sodium) availability on SNAT2 transcription and protein stability. Our findings reveal that the presence of extracellular sodium is crucial for inducing the transcriptional increase in SNAT2 gene expression in response to AA withdrawal and for maintaining expression and stability of SNAT2 protein.

## Materials and Methods

### Materials

Culture media [Dulbecco’s Modified Eagle’s Medium (DMEM)], fetal calf serum (FCS), and antibiotic/antimycotic solution were from Invitrogen (Paisley, United Kingdom). AAs and reagent grade chemicals were from Sigma–Aldrich (Poole, United Kingdom). Inhibitors were from Tocris (Bristol, United Kingdom) and CN Biosciences (Nottingham, United Kingdom).

### Molecular Biology, Cell Culture, and Transfection

Rat SNAT2 (Yao et al., 2000) and SNAT5 (Nakanishi et al., 2001) cDNAs were placed in pcDNA6 (Invitrogen) following PCR mutagenesis to introduce a C-terminal V5-His6 tag and a SNAT2–SNAT5 NcoI chimera generated in pCR2.1 and subcloned into pcDNA6 as described previously (Hyde et al., 2007). For some experiments, the rat SNAT2 promoter (including the first intronic element containing the tripartite AA responsive domain) was subcloned into pC3luc (Hyde et al., 2007). HeLa cells were transfected with pC3luc constructs (10 μg DNA/10 cm plate) using calcium phosphate. Luciferase activity was performed in 20 μg of cell lysate protein using the Luciferase Assay System (Promega, United Kingdom) according to the manufacturer’s instructions in a TD20/20 luminometer and was standardized relative to serum-starved, AA-supplemented cells.

HeLa cells were cultured in DMEM, supplemented with 10% FBS, and 1% (v/v) antibiotic/antimycotic solution (100 units/ml penicillin, 100 mg/ml streptomycin, and 250 ng/ml amphotericin B), at 37°C with 5% CO_2_. HeLa cells were incubated with HEPES-buffered saline [HBS; HEPES/NaCl (140 mM NaCl, 20 mM HEPES, 5 mM KCl, 2.5 mM MgSO_4_, 1 mM CaCl_2_, and 5 mM glucose, pH 7.4] supplemented with AA mix at 1× physiological concentration (Hyde et al., 2001) for 1 h, rinsed and incubated in the appropriate buffer for cell stimulation. In some experiments, Na^+^ salts in HBS buffer were replaced with equimolar concentrations of tetramethylammonium (TMA), *N*-methyl D-glucamine (NMDG), or choline salts.

HeLa cells were transiently transfected with pcDNA6-SNAT2^V5^, pcDNA6-SNAT5^V5^, pcDNA6-SNAT2-5^V5^ (10 μg DNA/10 cm plate or 3.5 μg DNA/well of six-well plate) using the polyethylenimine (PEI) method or in some experiments with pcDNA6-7A-SNAT2^V5^ [a sequence in which the seven lysine (K) residues in SNAT2 N-terminus were changed to alanine (A)] (3 μg DNA/6-cm plate) using lipofectamine. The 7A-SNAT2^V5^ sequence was synthesized by Life Technologies Geneart and the synthesized DNA was cloned into pcDNA6^V5^ plasmid *via* XhoI/XbaI digestion. It is important to stress that for all transfection experiments, comparative analysis of the effects that changes in substrate provision have upon cellular SNAT abundance was performed on cells cultured in plates/wells that were simultaneously transfected with the same V5-tagged construct and, consequently, in which transfection efficiency of plates/wells at the start of each experiment was the same (∼40–50%). As a result, any changes in SNAT-V5 protein abundance that occur in response to manipulating AA or Na^+^ availability is unlikely to be a consequence of variations in transfection efficiency between tissue culture plates/well.

### RNA Extraction, qPCR, and shRNA

Total RNA was extracted from HeLa cells using TRIzol^®^ reagent according to the manufacturer’s instructions (Sigma–Aldrich, Poole, United Kingdom). Quantitative real-time PCR was carried out using a StepOnePlus Real-Time PCR System (Applied Biosystems), SYBR Green JumpStart Taq ReadyMix (Sigma–Aldrich, Poole, United Kingdom), and primers targeting SNAT2 and SNAT5 with glyceraldehyde-3-phosphate dehydrogenase (GAPDH) being used as a control gene readout. The sequences for SNAT2, SNAT5, and GAPDH primers were as follows: *SNAT2*: forward, 5′-GCAGTGGAATCCTTGGGCTT-3′, SNAT2 reverse, 5′-ATAAAGACCCTCCTTCATTGGCA-3′; SNAT5: forward, 5′-GAGAGGGTGCCCGAACCT-3′, reverse, 5′-CCTCGAAATCCATGAACTGGAC-3′; GAPDH, forward, 5′-CCCCCGGTTTCTATAAATTGAGC-3′, reverse, 5′-GACCAAATCCGTTGACTCCGA-3′. PCR amplification was performed with an initial denaturation at 95°C for 10 min followed by 40 cycles of denaturation at 95°C for 15 s, annealing at 55°C for 15 s, and extension at 68°C for 1 min. The ratio of the SNAT2 or SNAT5 mRNA expression to that of GAPDH mRNA expression was calculated as described previously (Pfaffl, 2001).

The design and cloning of shRNA targeting Nedd4.2, lentiviral production, and generation of a stable Nedd4.2 silenced HeLa cell line was as previously described (Nardi et al., 2015).

#### Preparation of Total Cellular Membranes, Whole Cell Lysates, and Immunoblotting

Following appropriate experimental treatments as described in the text and figure legends, HeLa cells were rinsed twice with ice-cold phosphate-buffered saline then extracted on plates in lysis buffer containing 50 mmol/l Tris–HCl (pH 7.5), 1 mmol/l EDTA, 1 mmol/l EGTA, 1% (v/v) Triton X-100, 1 mmol/l Na_3_VO_4_, 10 mmol/l sodium-glycerophosphate, 50 mmol/l NaF, 5 mmol/l Na_4_P_2_O_7_, 1 μmol/l microcystin-LR, 0.27 mol/l sucrose, 0.2 mmol/l phenylmethylsulfonyl fluoride, 1 mmol/l benzamidine, 10 μg/ml leupeptin, and 0.1% (v/v) 2-mercaptoethanol. Total cell membranes were isolated from HeLa cells as described previously (Hajduch et al., 1998). Protein concentration in cell lysates and membranes was determined using the Bradford (1976) method. Proteins from membranes (10 μg) and whole cell lysates (30 μg) were separated on polyacrylamide resolving gels by SDS–PAGE. Proteins were transferred onto PVDF membrane by immunoblotting as described previously (Hajduch et al., 1998), and blocked with 5% (w/v) milk in 0.05% (v/v) Tween 20/Tris-buffered saline for 1 h. Membranes were then incubated overnight with primary antibodies [as indicated in figure legends: anti-Slc38a2 (SNAT2) was from MBL; anti-V5 was from Invitrogen; anti-GAPDH and anti-β-actin from Sigma; anti-Nedd4.2 was from Division of Signal Transduction and Therapy, University of Dundee; α-subunit of the Na,K-ATPase was from DSHB at the University of Iowa; anti-insulin receptor β-subunit was from Abcam], and then with an appropriate peroxidase-conjugated IgG for 1 h at room temperature. Immunoreactive bands were detected by enhanced chemiluminescence on Konica Minolta X-Ray film. Quantification of immunoblots was performed using ImageJ software^1^.

### Amino Acid Uptake

After experimental treatments, HeLa cells were incubated with 10 μmol/l [^14^C]Me-AIB (1 μCi/ml) for 10 min to assay Me-AIB uptake as previously described (Hajduch et al., 1998; Hyde et al., 2007). Radiotracer uptake over this time period was linear thus reflecting initial rates of uptake. Non-specific tracer binding was quantified by determining cell-associated radioactivity in the presence of a saturating dose of unlabeled Me-AIB (10 mM). To terminate uptake activity, cells were washed three times with isotonic saline (0.9% NaCl, w/v) and then lysed in 50 mM NaOH. Cell-associated radioactivity was determined by liquid scintillation counting and standardized to protein content determined using the Bradford (1976) method.

### Statistical Analyses

For multiple comparisons, statistical analysis was performed using one-way ANOVA. For individual comparisons, statistical analysis was performed using Student’s *t*-test. Data analysis was performed using GraphPad Prism software and considered statistically significant at *P* < 0.05.

## Results and Discussion

### Effects of Amino Acid Deprivation on SNAT2 Activity and Expression

**FIGURE 1:**
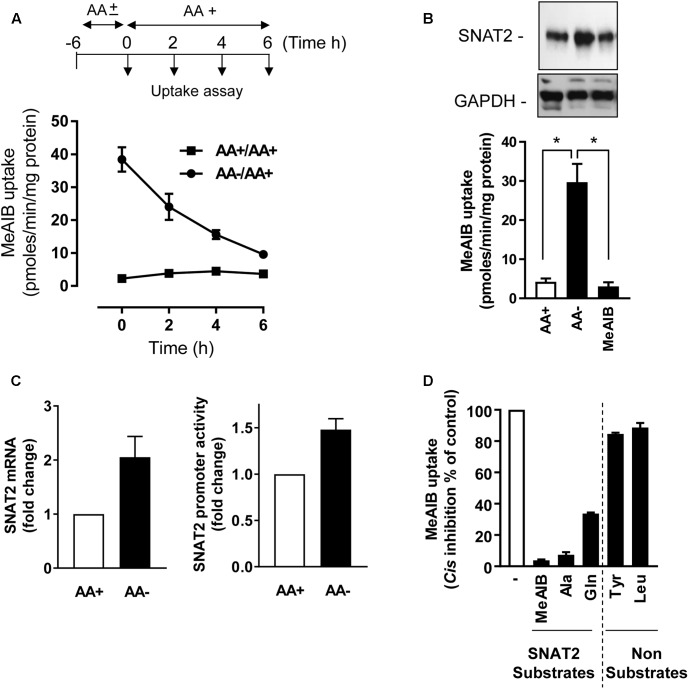
Effects of amino acid (AA) deprivation on Me-AIB uptake, SNAT2 mRNA, and SNAT2 protein abundance in HeLa cells. **(A)** HeLa cells were incubated in the absence or presence of 1× physiological AA mix for 6 h and then subsequently maintained or re-exposed to a 1× AA mix for times indicated prior to analysis of Me-AIB uptake. **(B)** Cells were incubated in the absence or presence of 1× physiological AA mix for 8 h or maintained in buffer in which the AA complement was substituted with 2 mM Me-AIB. Cells were subsequently either used for analysis of 10 μM Me-AIB uptake or lysed for analysis of SNAT2 protein abundance by immunoblotting with GAPDH used a loading control. **(C)** HeLa cells were transiently transfected with a pcDNA3 luciferase reporter construct, driven by the SNAT2 promoter; 48 h post-transfection cells were incubated in the absence (8 h) or presence of AAs. SNAT2 promoter activity was then determined by the analysis of luciferase activity (luminescence quantification). Results calculated as relative luminescence (arbitrary units) per microgram of protein and normalized as a fold change to a control value. Alternatively, following AA deprivation of cells SNAT2 mRNA was quantified using RT-PCR. **(D)** HeLa cells were AA deprived for 8 h and uptake of 10 μM [14C]-Me-AIB subsequently assessed in absence or presence of indicated AAs at 2 mM. Values are mean ± SEM from three to six separate experiments with each uptake measure being assayed in triplicate in individual experiments. The asterisks indicate a significant difference from indicated bars *P* ≤ 0.05.

In line with our previous work in L6 myotubes (Hyde et al., 2007), HeLa cells incubated in AA-free buffer exhibit a substantial adaptive increase in Me-AIB uptake compared with cells held in buffer containing a 1× physiological AA mix (**Figure 1A**). This increase in Me-AIB uptake declined rapidly with a *t*_1/2_ of ∼3–4 h upon re-exposing cells to AA-containing buffer. The adaptive increase in Me-AIB transport activity was associated with an increase in SNAT2 gene transcription (as judged by RT-PCR analysis of endogenous SNAT2 mRNA expression and increased activity from an ectopically expressed SNAT2-luciferase gene reporter construct) and an increase in native SNAT2 protein content (**Figures 1B,C**) that was repressed in cells incubated in buffer supplemented with Me-AIB alone (**Figure 1C**). As anticipated, the functional increase in [^14^C]-Me-AIB uptake that follows cellular adaptation to AA withdrawal was sensitive to *cis*-inhibition when uptake of the radiotracer was assayed in the presence of a competing concentration (2 mM) of unlabeled Me-AIB, Ala, and Gln (**Figure 1D**). All three AAs are established SNAT2 substrates suggesting that the measured increase in Me-AIB uptake reflects an increase in functional activity of the endogenous SNAT2 protein. Consistent with this suggestion neither Tyr nor Leu (whose cellular uptake is not mediated *via* SNAT2) had any significant inhibitory effect on the adaptive increase in [^14^C]-Me-AIB uptake (**Figure 1D**). Since Me-AIB is a non-metabolizable AA analog the above findings imply that the transcriptional-dependent increase in endogenous SNAT2 seen in AA-deprived cells is critically dependent upon whether SNAT2 is able to recognize/bind its substrate.

### Use of a SNAT2^V5^ Construct to Explore Substrate-Induced Changes in SNAT2 Stability

**FIGURE 2:**
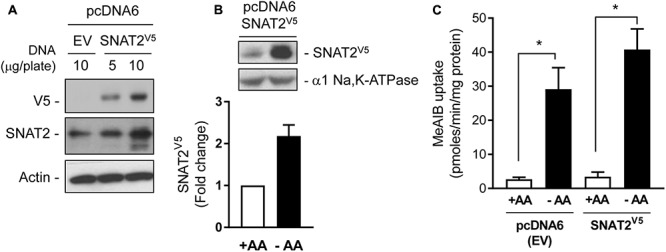
Expression of SNAT2^V5^ in HeLa cells. **(A)** HeLa cells were transiently transfected with 5 or 10 μg pcDNA6 either lacking (empty vector, EV) or containing SNAT2^V5^; 48 h post-transfection cells were lysed and whole cell lysates immunoblotted with antibodies to V5, SNAT2, and actin. **(B)** HeLa cells transiently expressing SNAT2^V5^ were incubated in the absence or presence of 1× physiological AA mix for 8 h and then subsequently lysed and immunoblotted using antibodies to V5 or the α1-subunit of the Na,K-ATPase (loading control). **(C)** Cells transfected with pcDNA6^V5^ (EV) or pcDNA6 containing SNAT2^V5^ were incubated in the absence or presence of 1× physiological AA mix for 8 h prior assaying Me-AIB uptake. Uptake values are mean ± SEM from three separate experiments, each conducted in triplicate. Asterisks indicate a significant difference between the indicated bars *P* ≤ 0.05.

The data presented in **Figure 1** are consistent with the idea that the increase in SNAT2 activity in AA-deprived cells involves a transcriptional response. However, we have also shown previously that under these circumstances SNAT2 protein can be stabilized post-translationally (Hyde et al., 2007), but what remains unclear at present is whether this enhanced stability is regulated in a substrate-dependent manner. To this end, we transiently expressed a CMV-driven SNAT2 construct (containing a C-terminal exofacial V5-His_6_ epitope tag) in a pcDNA6 vector in HeLa cells. The CMV promoter lacks the AA-responsive domain present in the SNAT2 gene and consequently the SNAT2^V5^ transporter is expressed constitutively and any changes in its abundance reflect changes in SNAT2 protein stability alone. **Figure 2A** shows that a V5 immunoreactive band could be detected in lysates of HeLa cells transfected with 5 or 10 μg of pcDNA6 containing the CMV-driven SNAT2^V5^ construct, but not in cells transfected with the empty vector. The increased expression of SNAT2^V5^ in transfected cells could also be detected using an anti-SNAT2 antibody that binds to the endogenous and the V5-tagged transporter. **Figure 2B** shows that while SNAT2^V5^ was detectable in HeLa cells transfected with pcDNA6–SNAT2V5 when maintained in AA sufficient media, the abundance of this ectopically expressed transporter was elevated by approximately two-fold when cells were AA-deprived for 8 h. Since expression of SNAT2^V5^ is driven by an AA insensitive CMV promoter and transfection efficiency between cells incubated in buffer containing or lacking AAs was similar within each experiment, the elevated SNAT2^V5^ abundance induced upon AA deprivation is likely to reflect increased stabilization of the carrier. This latter finding is consistent with previous work from our lab (Hyde et al., 2007, 3143 /id), but we show here that the increase in SNAT2^V5^ is also associated with a modest (∼30%) increase in Me-AIB uptake over and above that recorded in control cells transfected with the empty vector, in which, the elevated Me-AIB uptake elicited by AA deprivation is due to the adaptive upregulation of the endogenous SNAT2 transporter (**Figure 2C**).

**FIGURE 3:**
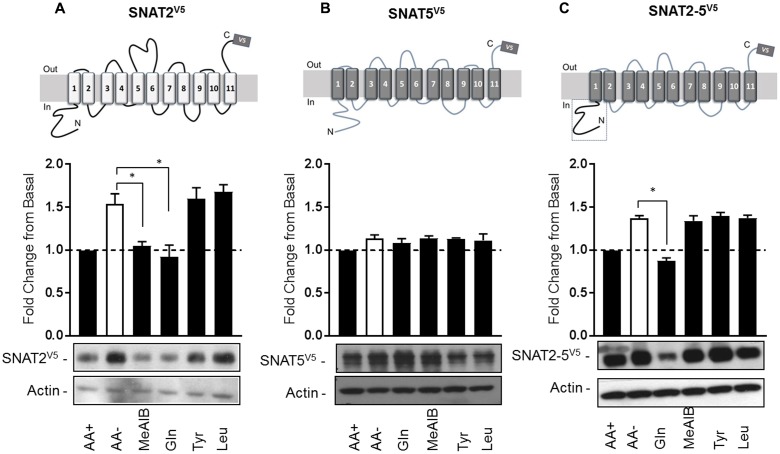
Substrate-induced regulation of SNAT2 stability. HeLa cells were transiently transfected with 10 μg of a pcDNA6 vector containing **(A)** SNAT2^V5^, **(B)** SNAT5^V5^, or **(C)** SNAT2-5^V5^; 48 h post-transfection, cells were incubated for 8 h in the absence or presence of AAs or in EBSS containing 2 mM of the indicated AA. Lysates (40 μg) were then subject to SDS–PAGE and immunoblotting with an anti-V5 antibody or antibody directed against actin, which was used as a gel loading control. Blots are representative of three separate experiments, bar graphs values are mean ± SEM. V5 expression was quantified using ImageJ software and results expressed as a fold change in expression of the transporter relative to that in cells incubated in the presence of AAs. Asterisks indicate a significant difference between the indicated bars (*P* ≤ 0.05).

Having established that SNAT2^V5^ is expressed and stabilized within AA-deprived HeLa cells, we subsequently assessed whether this increase in protein stability was sensitive to the presence of SNAT2 substrates/non-substrates. HeLa cells were transiently transfected with the CMV-driven SNAT2^V5^ construct and subsequently incubated in the absence and presence of Me-AIB, Gln, Tyr, or Leu during the AA deprivation period. **Figure 3A** shows that compared with cells maintained in buffer containing a 1× AA mix, SNAT2^V5^ protein abundance increased by ∼1.6-fold upon incubating cells for 8 h in buffer lacking AAs. Strikingly, this increase in SNAT2^V5^ protein was repressed if cells were held in buffer containing 2 mM Me-AIB or Gln alone, but not if these AAs were substituted with either Tyr or Leu. This observation indicates that only SNAT2 substrates repress the enhanced stability of SNAT2^V5^ under circumstances when the full physiological AA complement is otherwise absent.

Unlike SNAT2, SNAT5 [a closely related SLC38 member of the System N family (Mackenzie and Erickson, 2004)] does not exhibit AA-regulated transcriptional upregulation nor is the stability of this transporter modified post-translationally (Hyde et al., 2007). Consistent with these previous observations, **Figure 3B** shows that SNAT5^V5^ protein abundance was not altered significantly when cells expressing this construct were deprived of AAs or maintained in buffer containing the indicated AAs. We have previously shown that the increase in SNAT2 stability seen upon AA withdrawal is associated with an isoform-specific regulatory domain(s) present within the hydrophilic N-terminal region, because “substituting” the corresponding N-terminal region of SNAT5 with that of SNAT2 stabilized the chimeric SNAT2-5^V5^ protein upon AA withdrawal (Hyde et al., 2007; **Figure 3C**). Intriguingly, this stabilization can be repressed by resupply of Gln, but not Me-AIB, Tyr, or Leu (which importantly are not SNAT5 substrates). This observation indicates that the N-terminal domain of SNAT2 confers a stabilization signal in the absence of substrates and significantly, that transporter stability is mechanistically linked to substrate binding and/or translocation.

### SNAT2 Stability Is Not Regulated by Nedd4.2 but Is Dependent on Putative Lysyl Ubiquitination Residues within the N-Terminal Tail

**FIGURE 4:**
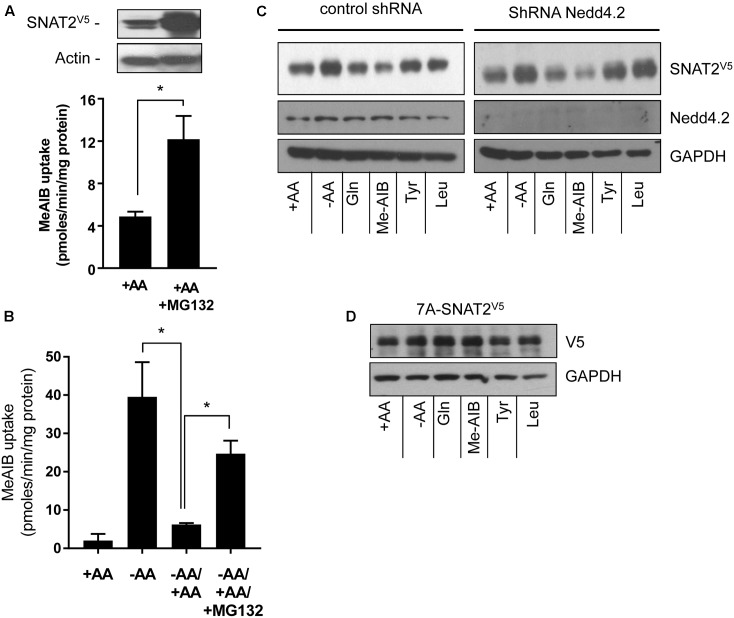
Effects of the proteasomal inhibitor, MG132, and Nedd4.2 gene silencing on SNAT2 stability, and Me-AIB uptake in HeLa cells. **(A)** HeLa cells were transiently transfected with 10 μg of a pcDNA6 vector containing SNAT2^V5^ and incubated in buffer containing AAs but lacking or supplemented with MG132 (10 μM) for 8 h. After this period cells were either lysed for analysis of SNAT2^V5^ by immunoblotting or used to assay Me-AIB uptake. **(B)** HeLa cells were incubated in the absence or presence of 1× physiological AA mix for 8 h and then subsequently maintained or re-exposed to a 1× AA mix 6 h in the absence or presence of 10 μM MG132 prior to assaying Me-AIB uptake. Bar values (A and B) are mean ± SEM from three to five separate experiments with experiment conducted in triplicate. Asterisks indicate a significant difference between indicated bars *P* ≤ 0.05. **(C)** HeLa cells stably expressing shRNA targeting Nedd4.2 or a control shRNA (Scr) were incubated for 8 h in the absence or presence of AAs or in EBSS containing 2 mM of the indicated AA. Lysates (40 μg) were then subject to SDS–PAGE and immunoblotting with an anti-V5 antibody or antibodies directed against Nedd4.2 or GAPDH, which was used as a gel loading control. **(D)** HeLa cells were transiently transfected with 7KA-SNAT2V5 mutant, 24 h post-transfection cells were treated as in **(C)**, and lysates immunoblotted with an anti-V5 antibody or GAPDH antibody. The blot is representative of two similar experiments.

The proteasome inhibitor MG132 has been shown to increase SNAT2 transport activity in 3T3-L1 adipocytes under AA sufficient conditions, suggesting that steady-state levels of the transporter may be influenced by the ubiquitin-proteasome system (UPS) (Hatanaka et al., 2006). Indeed, the ubiquitin E3-ligase Nedd4.2 has been implicated as a regulator of SNAT2 ubiquitination (Hatanaka et al., 2006). To assess whether proteasomal activity features in the substrate-induced regulation of SNAT2 transporter stability, we assessed what impact MG132 has upon SNAT2-mediated transport under (i) AA sufficient conditions and (ii) upon resupply of AAs to cells that had been subject to a period of AA deprivation. **Figure 4A** shows that HeLa cells transiently transfected with SNAT2^V5^ exhibit greater SNAT2^V5^ protein expression under AA-sufficient conditions when incubated with 10 μM MG132 for 8 h than those exposed to vehicle solution alone and also that this coincides with a significant increase in Me-AIB uptake. In line with the data shown in **Figure 1A**, in normal untransfected HeLa cells, AA withdrawal for an 8-h period induced a robust adaptive increase in Me-AIB uptake, which was repressed to near base line values upon AA resupplementation for 6 h (**Figure 4B**). However, this repression was significantly blunted if MG132 was present during the AA resupply period (**Figure 4B**). This observation implies that proteasomal inhibition with MG132 may restrain degradation of endogenous SNAT2 and, by doing so, help preserve a greater component of the SNAT2-mediated transport activity that is otherwise rapidly lost upon resupplying AAs to cells previously subjected to AA deprivation. In support of this idea, we recently reported that SNAT2 exhibits greater ubiquitination when AAs are present in the culture buffer and is thus more likely to be degraded by the UPS under these circumstances then when AAs are absent. Rapid turnover of SNAT2 protein involving the UPS would therefore help maintain relatively low levels of SNAT2 protein and transport activity in AA-replete cells (Nardi et al., 2015). Since MG132 is unlikely to curtail internalization and lysosomal-directed degradation of SNAT2 carriers from the cell surface, it is not surprising that the inhibitor does not completely attenuate the AA-induced repression of SNAT2 transport activity (**Figure 4B**).

To test whether Nedd4.2 participates in the substrate-induced destabilization of SNAT2, we generated HeLa cells in which Nedd4.2 had been stably silenced using a lentiviral shRNA strategy (Nardi et al., 2015). **Figure 4C** shows that HeLa cells exhibit enhanced stabilization of SNAT2^V5^ upon AA deprivation which was repressed in a substrate-dependent manner irrespective of whether Nedd4.2 was present or if its expression had been substantially depleted by gene silencing; an observation that appears to exclude Nedd4.2 as a regulator of SNAT2 stability in our cell system. Since “grafting” the N-terminal domain of SNAT2 onto SNAT5 bestows substrate-dependent changes in stability upon a SNAT2–5 chimeric protein, we postulated that this domain was most likely targeted for ubiquitination by an unidentified E3-ligase. Ubiquitination of substrate proteins is canonically targeted at lysyl residues and it is noteworthy that a number of cell surface transporters [e.g., epithelial Na^+^ channel (ENaC) and neurotransmitter transporters including those for AAs such as γ-aminobutyric acid (GABA), glutamate, and glycine] are ubiquitinated on lysyl residues within their N-terminal domains (Staub et al., 1997; Sheldon et al., 2008). Ubiquitination of cell surface proteins is recognized as a common mechanism promoting internalization and endosomal sorting that results in either their recycling back to the plasma membrane or targeting to the proteasome or lysosome for degradation [reviewed in Miranda and Sorkin (2007)]. Much like the above transporters, the N-terminal domain of SNAT2 possesses seven putative lysyl ubiquitination targets. We therefore expressed a SNAT2^V5^ construct in which all seven of these lysyl residues were mutated to alanine (7A-SNAT2^V5^). **Figure 4D** shows that, unlike wild-type SNAT2 (**Figure 4C**), the stability of the 7A-SNAT2^V5^ protein is not modified by changes in AA availability nor is it sensitive to substrate-induced destabilization. Since the 7A-SNAT2^V5^ protein is equally stable under the different conditions tested, we argue that binding of AA substrates to the native transporter may induce a conformational change in the protein that make one or more of the target lysyl residues in the N-terminal SNAT2 domain accessible for ubiquitination by its E3-ligase that then promotes ubiquitinated SNAT2 internalization/degradation.

### The Role of Na^+^ in Regulating Transcriptional and Post-translational Aspects of the SNAT2 Adaptive Response

**FIGURE 5:**
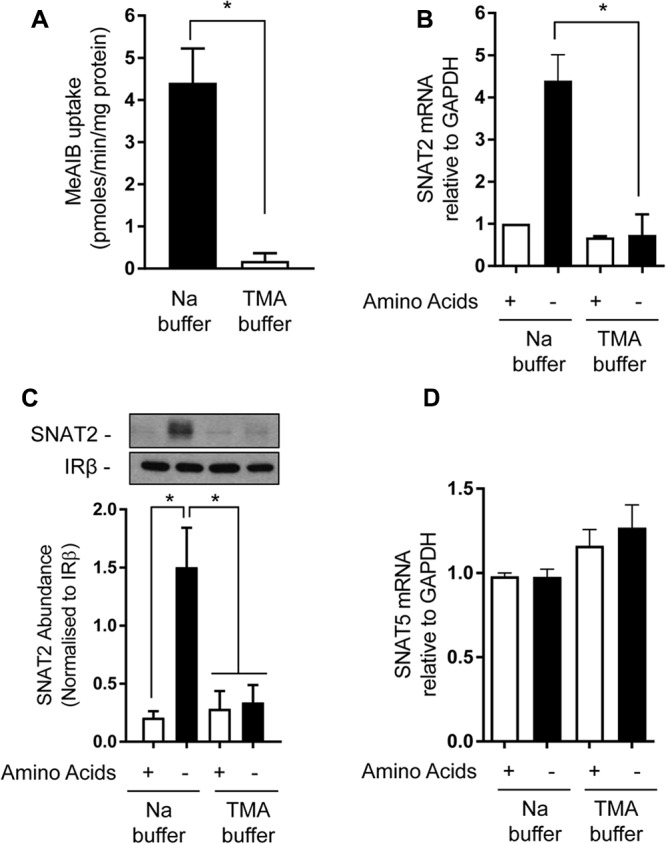
Effects of extracellular sodium replacement on Me-AIB uptake, SNAT2, and SNAT5 gene expression. **(A)** Basal Me-AIB uptake was assayed in HeLa cells using uptake buffer containing a physiological Na concentration or one in which the cation was replaced with an equimolar concentration of TMA. **(B–D)** HeLa cells were cultured in DMEM media and subsequently incubated for 8 h in EBSS or buffer in which Na had been replaced with an equimolar concentration of TMA that contained or lacked a 1× physiological AA mix. Cells were subsequently washed and harvested for analysis of SNAT2, SNAT5, and GAPDH gene expression by quantitative PCR analysis of RNA **(B,D)** or analysis of SNAT2 protein **(C)** in total membranes isolated from cells. Data in bar graphs are presented as mean ± SEM (*n* = 3) with asterisks indicating significant changes (*P* < 0.05) between the indicated bars.

Amino acid uptake *via* SNAT2 is critically dependent upon the availability of Na^+^ as a co-substrate and previous studies have identified helices 1 and 8 as being important for accommodating the Na^+^ binding site, given that mutation of Asn82 and Thr384, respectively, located within these helices reduces the transporter’s affinity for Na^+^ and its capacity for mediating AA transport (Zhang et al., 2008, 2009). However, to our knowledge, the importance of Na^+^ as a determinant of the SNAT2 adaptive/stability response has not been previously addressed. In line with the importance of Na^+^ as a co-substrate, replacement of extracellular NaCl with TMACl was found to virtually abolish cellular Me-AIB uptake (**Figure 5A**). As anticipated, subjecting HeLa cells to AA deprivation for 8 h induced a significant increase in mRNA abundance of the endogenous SNAT2 transporter, but this was not seen when cells were AA deprived for this period in TMACl-containing buffer (**Figure 5B**). In line with this observation, analysis of native SNAT2 protein expression revealed a strong increase in the abundance of the endogenous transporter when cells were AA deprived, but this response was absent if cells were AA deprived in buffer also lacking Na^+^ (**Figure 5C**). In contrast, the transcriptional expression of endogenous SNAT5, a related Na^+^-coupled AA transporter, was unaltered in response to AA insufficiency and was marginally, but not significantly, elevated in response to extracellular Na^+^ withdrawal (**Figure 5D**).

**FIGURE 6:**
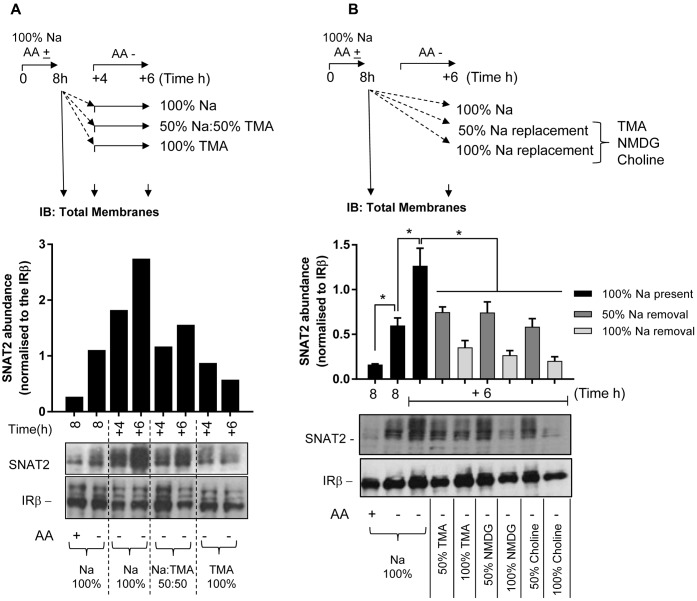
Effects of changes in extracellular sodium on native SNAT2 protein expression in HeLa cells. **(A)** HeLa cells were maintained in the absence or presence of a 1× physiological AA mix for 8 h in HEPES buffer (HBS) containing 140 mM NaCl (Na-HBS). Cells were either harvested or incubated for a further 4 or 6 h in AA-deficient Na-HBS, or HBS in which NaCl was replaced with 50 or 100% TMACl. At the end of these periods cells were harvested and total membranes (10 μg) prepared, which were subjected to SDS–PAGE and were then immunoblotted with antibodies against SNAT2 or the beta-subunit of the insulin receptor (IRβ). **(B)** HeLa cells were incubated for an initial 8 h with or without a 1× AA mix in Na-HBS and then harvested or incubated for an additional 6 h without AAs in a partial (50%) sodium-replaced, or fully (100%) sodium-replaced HBS media. Sodium was partially or fully replaced with either TMA, NMDG, or choline. At the end of the indicated incubation periods indicated, cells were harvested and total membranes (10 μg) prepared before SDS–PAGE and immunoblotting against SNAT2 and IRβ (used as a loading control). Endogenous SNAT2 expression was quantified using ImageJ software and results expressed as relative abundance with respect to the IRβ. Blots are representative of three separate experiments, bar graphs values are mean ± SEM (*n* = 3). Asterisks indicate a significant difference between the indicated bars (*P* ≤ 0.05).

Following these observations, we subsequently assessed the effects of removing extracellular Na^+^ upon protein expression of the native SNAT2 transporter expressed in HeLa cells using two different strategies. The first involved subjecting HeLa cells to an initial 8 h period of AA deprivation in media containing a physiological Na^+^ concentration (140 mM). At the end of this period, cells were either harvested (for isolation of total cell membranes) or maintained for a further period (4 or 6 h) in AA-free buffer in which Na^+^ was present at 100, 50, or 0% of its physiological concentration (using TMA as the cation replacement). At the end of each of these additional incubation periods, cells were harvested and total membranes isolated and the abundance of endogenous SNAT2 determined by immunoblotting. **Figure 6A** shows that 8 h of AA deprivation induced a notable elevation in native SNAT2 protein abundance, which was further elevated in a time-dependent manner if cells were subjected to an extended period of AA deprivation in Na^+^-containing buffer for up to 14 h. However, this increase in endogenous SNAT2 was blunted if the AA-free media during the extended period contained 50% of the physiological Na^+^ concentration and absent if the Na^+^ was completely replaced with TMA. In contrast to effects on SNAT2, manipulating the AA or Na^+^ content of the extracellular buffer had no detectable effect on the membrane abundance of the insulin receptor beta-subunit, which was used as a gel loading control (**Figure 6A**). To exclude the possibility that these effects on endogenous SNAT2 protein abundance were induced by TMA through a mechanism that acts independently of its role as a Na^+^ cation replacement, we also assessed the effects of using NMDG and choline as Na^+^ ion replacements. As with TMA, **Figure 6B** shows that replacing Na^+^ with either of these two cations led to a concentration-dependent reduction in native SNAT2 protein abundance despite cells being maintained in AA-deprived buffer.

The decline in SNAT2 protein seen upon incubating cells in an environment with reduced Na^+^ may not just reflect a failure to sustain the adaptive increase in SNAT2 gene expression under AA-insufficient conditions, but also a reduction in SNAT2 protein stability. To assess whether Na^+^ may be an important determinant of SNAT2 stability, two additional experimental strategies were employed.

**FIGURE 7:**
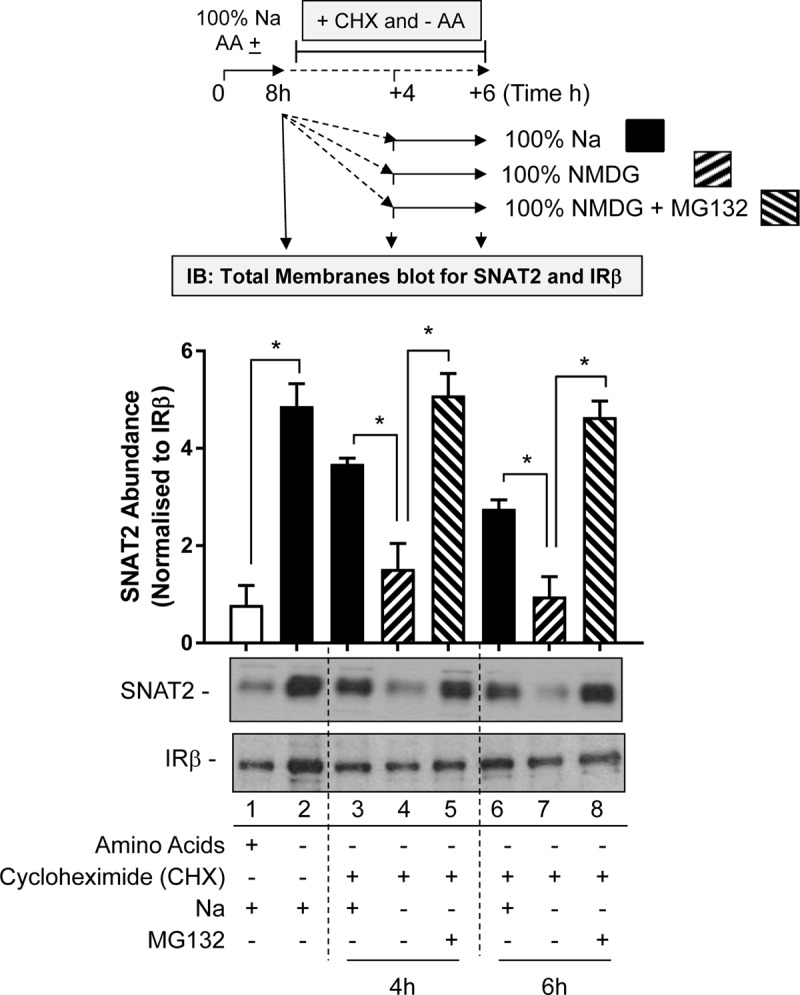
Effects of Na replacement and MG132 on endogenous SNAT2 protein stability. HeLa cells were maintained in the absence or presence of a 1× physiological AA mix for 8 h in HBS containing 140 mM NaCl (Na-HBS). Cells were either harvested or incubated for a further 4 or 6 h in AA-deficient buffer containing cycloheximide (50 μg/ml). This buffer either contained 140 mM Na^+^ or the cation was fully replaced with NMDG or was replaced with buffer containing NMDG supplemented with MG132 (10 μM). At the end of the initial 8 h or the extended 4 and 6 h AA deprivation periods cells were harvested and total membranes prepared and 10 μg membrane protein subjected to SDS–PAGE prior to immunoblotting with antibodies against native SNAT2 or the beta-subunit of the insulin receptor (IRβ). Endogenous SNAT2 expression was quantified using ImageJ software and results expressed as relative abundance with respect to the IRβ. Blots are representative of three separate experiments, bar graphs values are mean ± SEM. Asterisks indicate a significant difference between the indicated bars (*P* ≤ 0.05).

The first involved subjecting HeLa cells to an 8-h period of AA deprivation to induce the characteristic adaptive increase in SNAT2 protein expression. After this period, cells were washed and incubated for a further 4 or 6 h in AA-free buffer containing cycloheximide (CHX) so as to block further synthesis of native SNAT2 protein that is driven by the increased expression of the endogenous SNAT2 gene. During this extended AA deprivation period the incubation buffer either contains Na^+^ at a physiological concentration or was fully replaced with NMDG. At the end of the extended 4 or 6 h AA deprivation period, total membranes from HeLa cells were prepared and endogenous SNAT2 protein content assessed by immunoblotting. **Figure 7** shows that AA deprivation for 8 h induces a robust increase in native SNAT2 abundance (compare lanes 1 and 2), but that this subsequently declines if synthesis of new SNAT2 protein was halted by CHX despite the absence of AA in the buffer (compare lanes 2, 3, and 6). This decline most likely reflects degradation of endogenous SNAT2 and our data indicate that this loss was significantly accelerated if extracellular Na^+^ was replaced with NMDG (compare lanes 3 and 4; lanes 6 and 7). This proposition is further strengthened by the demonstration that we can attenuate the rapid loss in native SNAT2 protein induced by extracellular Na^+^ removal by supplementing the Na-free buffer with the proteasomal inhibitor, MG132 (compare lanes 4 and 5; lanes 7 and 8).

**FIGURE 8:**
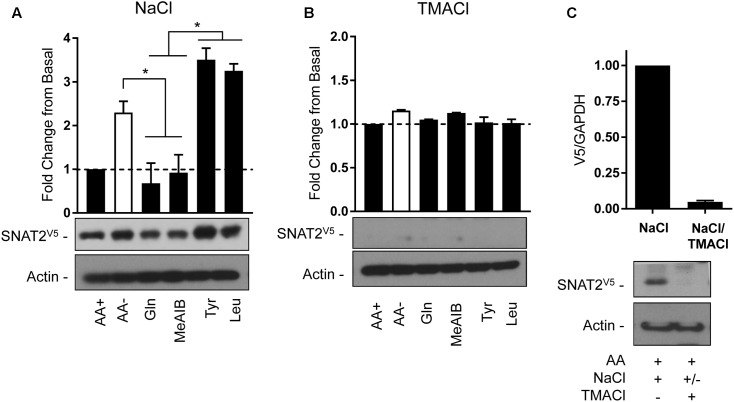
Effects of changes in extracellular AA availability and sodium on transiently expressed SNAT2^V5^ protein stability in HeLa cells. HeLa cells were transiently transfected with 10 μg of a pcDNA6 vector containing SNAT2^V5^ and subsequently incubated in **(A)** HBS containing 140 mM NaCl or **(B)** buffer in which NaCl had been replaced with TMACl for 8 h in the absence or presence of AAs or with the appropriate HBS or TMACl buffer containing 2 mM of the indicated AA. **(C)** Forty-eight hours post-transfection a population of SNAT2^V5^ transfected cells maintained in AA-containing HBS buffer was either lysed or subsequently washed “free” of HBS and then incubated for a further 6 h in AA-containing buffer in which NaCl had been replaced with TMACl prior to lysis. Lysates **(A–C)** (40 μg) were then subject to SDS–PAGE and immunoblotting with an anti-V5 antibody or antibody directed against actin, which was used as a gel loading control. Blots are representative of three separate experiments, bar graphs values are mean ± SEM. V5 expression was quantified using ImageJ software and results expressed as a fold change in expression of the transporter relative to that in cells incubated in the presence of AAs. Asterisks indicate a significant difference between the indicated bars (*P* ≤ 0.05).

The second strategy utilized the transient expression of SNAT2^V5^ in HeLa cells. In line with the data presented in **Figure 3A**, depriving cells of AAs in buffer containing a physiological Na^+^ concentration induced a significant increase in SNAT2^V5^ stability, which was not observed if Gln or Me-AIB (SNAT2 substrates) were present in the buffer whereas presence of Tyr or Leu (non-substrates) did not restrain the increased stabilization of the transporter (**Figure 8A**). Strikingly, expression of SNAT2^V5^ or evidence of its stabilization in response to AA deprivation was not evident if cells transfected with the pcDNA6–SNAT2^V5^ were incubated in TMACl-containing buffer for 8 h prior to immunoblot analysis (**Figure 8B**). While it is possible that extracellular Na^+^ replacement may have suppressed expression of the CMV-driven SNAT2^V5^ construct, we considered it was more likely that replacement of extracellular Na^+^ from cells in which the transporter construct had initially been expressed under Na^+^ replete conditions may have led to rapid degradation of the transporter protein. To test this, cells transfected with pcDNA6–SNAT^V5^ in Na^+^ containing buffer were split into two subpopulations 48 h post-transfection. One population was immediately lysed and immunoblotted to confirm the expression of the V5-tagged transporter, while the other was washed and subsequently maintained, for a further 6 h, in buffer in which sodium had been replaced with TMA prior to immunoblotting. **Figure 8C** shows that while SNAT2^V5^ was readily detectable in the first population of cells that were held in Na^+^-containing buffer, the transporter was barely detectable in the second population of cells (from the same transfection experiment) when subsequently incubated in buffer in which Na^+^ was completely replaced by TMA. Both endogenous SNAT2 and transiently overexpressed SNAT^V5^ exhibit similar protein instability in the absence of Na^+^. Based on these observations it might have been anticipated that previous studies in which SNAT2 residues implicated in Na^+^ binding (Asn82 and Thr384) had been mutated may have reported reduced expression/stability of the carrier. However, immunofluorescence and cell surface biotinylation analysis of SNAT2 harboring mutations at these residues suggests that membrane expression of the mutated transporter is maintained (Zhang et al., 2008, 2009). Since mutation of Asn82 and Thr384 is associated with reduced carrier affinity rather than loss in Na^+^ binding *per se*, it is possible that despite the reduced cation affinity, the presence and binding of Na^+^ may be sufficient to help sustain SNAT2 expression/stability. We speculate that the binding of Na^+^ to SNAT2 (endogenous or ectopically expressed) in the cell membrane imposes a stabilized (N-terminal tail occluded) conformation and that, in the additional presence of AA, substrate-induced cycling of the transporter periodically exposes the N-terminus for ubiquitination when substrates are released and thus reduces protein stability. In the absence of either Na^+^ or AA substrates, the exposed N-terminal conformation maximizes the likelihood of SNAT2 protein ubiquitination and subsequent proteolysis.

## Conclusion

Collectively, the findings presented herein imply that while SNAT2-mediated AA uptake is substantially reduced in Na^+^-free buffer; a scenario that would essentially mimic a state of AA deprivation, sensing of SNAT2 substrate insufficiency and its coupling to the transcriptional activation of the SNAT2 gene and to processes regulating carrier stability is crucially dependent on the presence and perhaps binding of extracellular Na^+^ to the transporter. While these observations further strengthen the concept that SNAT2 possesses transceptor-like functions, precisely how substrate occupancy/transport is mechanistically linked to molecules that regulate its cellular expression and protein turnover is unclear and remains important investigative goals of future work.

## Author Contributions

TH, EC, CS, RH, and DS participated in the study design, performed the research, and conducted the data analysis. PT and HH participated in the study design/data analysis, wrote, and edited the manuscript.

## Conflict of Interest Statement

The authors declare that the research was conducted in the absence of any commercial or financial relationships that could be construed as a potential conflict of interest.

## Footnotes

1 http://rsbweb.nih.gov/ij/
